# Optimal timing and capacity choice under the rate-of-return renewable energy support

**DOI:** 10.1016/j.mex.2020.100828

**Published:** 2020-02-24

**Authors:** Mariia Kozlova, Stein-Erik Fleten, Verena Hagspiel

**Affiliations:** a School of Business and Management, Lappeenranta University of Technology, Finland; b Department of Industrial Economics and Technology Management, Norwegian University of Science and Technology, Norway

**Keywords:** Dynamic programming, Real options, Renewable energy policy, Investment timing, Capacity choice

## Abstract

This article presents a stylized renewable energy (RE) investment project profitability analysis under a rate-of-return RE support type. We use a dynamic programming approach to value the real options. While the method is widely used in RE policy analysis, the rate-of-return support is presented in this framework for the first time. We formulate a stylized RE project under the rate-of-return regulation in the dynamic programming framework and solve for optimal investment timing and project size.

• A stylized renewable energy (RE) investment under rate-of-return RE support is presented in the dynamic programming framework;
• The system is solved for optimal capacity choice in the presence of the electricity price uncertainty. We also comment on the optimal investment timing, which turns out to be a now-or-never decision in this case.

## Graphical abstract

**Figure:**
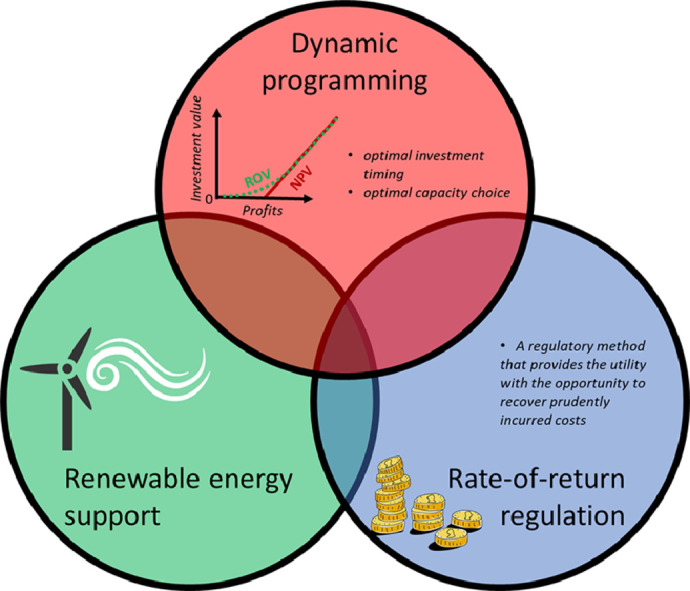

**Table:** Specification Table

Subject Area:	*Social Sciences*
More specific subject area:	*Financial analysis of renewable energy support*
Method name:	*Dynamic programming*
Name and reference of original method:	*The original method is presented in* [1] Dixit, A. K. & Pindyck, R. S., *Investment under uncertainty*. Princeton university press, 1994
Resource availability:	*The context, details and discussion of the results can be found in* [2] Kozlova M., Fleten S.–E., Hagspiel V., An Alternative Design of Renewable Energy Support: Investment Timing and Capacity Choice under the Russian Capacity Mechanism, Energy, 174, 2019, 591–601

## Method details

The method is based on the well-defined real options dynamic programming approach [1], applied in the context of RE investment, as in e.g. [3], and adjusted to describe a new type of RE support [2].

A RE investment project is assumed to be characterized by the following investment cost function (1) and electricity production (2)

(1) I(x)=Ax,

where *A* is capital costs (EUR/GW) unit of capacity installed, and *x* is the installed capacity (GW).

(2) $$Q\left(x\right)=ax^{b}\;\text{with}\;a>0\;\text{and}\;0\;<b<1$$

where *a* is capacity factor (hours per year), and *b* is a unit-less parameter of the production function that can be interpreted as a wake effect. It reflects that the more capacity is installed at a given licensed site, the less productive these units will be. For the case of wind (see, for example, [4]) it simply reflects that there is less wind behind an operating wind mill. Placing two mills close to each other will reduce the output per unit of capacity.

The rate-of-return subsidy is defined as an annuity based on project investment costs *I*(*x*), plus operating costs, and minus expected revenues from electricity sales *Q*(*x*)*S*(*t*), where *S*(*t*) is the stochastic electricity price. “Expected revenues”, in this context, reflects that the subsidy is not calculated only once, but is recalculated over time for each individual project. Further, the investment cost part is corrected by a coefficient that reflects project electricity production performance *k*(*Q*(*x*)). In a simplified form, assuming an infinite lifetime of the project and neglecting operating costs, the capacity payments of such a return regulation (*RR)* can be represented as

(3) RR(S(t);x)=I(x)Rk(Q(x))−Q(x)S(t)

where *R* is the return on investment provided by the subsidy,

*I*(*x*)*R* represents the perpetual annuity payments (the core idea of the rate-of-return subsidy).

The electricity production performance coefficient *k*(*Q*(*x*)) depends on the production performance (*Q*(*x*)). *k* is defined as

(4) $$k\left(Q\left(x\right)\right)=min\left(1,\frac{Q\left(x\right)}{Q_{target}}\right)$$

where *Q_target_* is the target electricity production level set by the subsidy.

The profit flow of the project under the rate-of-return subsidy consists of electricity sales and the subsidy payments

(5) $$\Pi_{RR}\left(S(t);x\right)=Q\left(x\right)S\left(t\right)+RR\left(S(t);x\right)$$

Since the subsidy payments are set to account for revenue from electricity sales (3), this term cancels out making the profit flow independent of stochastic electricity prices

(6) $$\Pi_{RR}\left(x\right)=I\left(x\right)Rk\left(Q(x)\right)$$

According to the Bellman equation, the return on the project (or option) is equal to instantaneous profits plus the expected appreciation of the project value

(7) $$\rho V=\Pi +\frac{1}{dt}E\left(dV\right)$$

where *V* is the project (or option) value, and *ρ* is the discount rate.

Since uncertainty in profits under the rate-of-return subsidy is eliminated, the profit flow (6) is fully deterministic^1^. The real option value is then equal to the maximum of the net present value (NPV) and zero. This means that the optimal time for investment is either now or never. Immediate investment is optimal if NPV > 0, where the project value upon investment is equal to the discounted future profits

(8) $$V_{RR}\left(x\right)=\int_{0}^{\infty }I\left(x\right)k\left(Q\left(x\right)\right)R\;e^{-\rho s}ds=I\left(x\right)k\left(Q\left(x\right)\right)\frac{R}{\rho }$$

From this formulation, it can be observed that in case of sufficient electricity production performance (k(Q(x))=1), the value of the project is defined by the ratio of the provided subsidy interest rate to the actual discount rate^1^

(9) $$V_{RR}\left(x\right)=\left\{ \begin{array}{cc} I\left(x\right)\frac{R}{\rho }, & if\;k\left(Q\left(x\right)\right)=1\\ I\left(x\right)k\left(Q\left(x\right)\right)\frac{R}{\rho }, & if\;k\left(Q\left(x\right)\right)<1 \end{array}\right.$$

The project NPV is equal to the discounted future profit flows defined in (6) minus the investment cost

(10) $$NPV_{RR}\left(x\right)=I\left(x\right)k\left(Q\left(x\right)\right)\frac{R}{\rho }-I\left(x\right)$$

or

(11) $$NPV_{RR}\left(x\right)=I\left(x\right)\left(k\left(Q\left(x\right)\right)\frac{R}{\rho }-1\right)$$

The subsidy is calculated based on the planned investment cost declared in the auction bid. However, the actual investment cost can be different because of, for example, overspending due to a change in contractors. In this case (10) is reformulated to

(12) $$NPV_{RR}\left(x\right)=I{\left(x\right)}_{planned}k\left(Q\left(x\right)\right)\frac{R}{\rho }-I{\left(x\right)}_{realized}$$

where *I*(*x*)_*planned*_ is the stated investment cost in the auction bid, and is taken into the subsidy calculation, and *I*(*x*)_*realized*_ is the actual realized investment cost.

Eq. (12) highlights the fact that if there is unexpected overspending, the subsidy payments would not compensate it. If $I{\left(x\right)}_{planned}=I{\left(x\right)}_{realized}$, the net payoff of a project under the rate-of-return subsidy is defined by (11).

Two cases should be considered for the optimal capacity choice: first, when project electricity production is expected to be less than the set target and second (the coefficient (4) is less than 1), when it is equal to or higher than the target (the coefficient (4) is equal to 1). The target production is defined as the target capacity factor multiplied by the installed capacity

(13) $$Q_{target}=a_{target}x$$

In the first case, when project production is less than the target, *Q*(*x*) < *Q_target_*, the coefficient (4) is equal to

(14) $$k\left(Q\left(x\right)\right)=\frac{Q\left(x\right)}{Q_{target}}=\frac{ax^{b}}{a_{target}x}=\frac{a}{a_{target}}x^{b-1}$$

The optimal capacity can be found by equating the marginal present value to the marginal investment cost (Boomsma et al., 2012)

(15) $$\frac{dV}{dx}\left(x^{*}\right)=\frac{dI}{dx}$$

Plugging in the present value (8) with the coefficient (14) and the investment cost (1), taking the derivative and solving for x* we arrive at the following formulation of the optimal capacity

(16) $$x^{*}={\left(\frac{ab}{a_{target}}\frac{R}{\rho }\right)}^{\frac{1}{1-b}}$$

Eq. (16) shows that the optimal capacity increases in the production function parameters *a* and *b*, and decreases in the discount rate *ρ*.

In the second case, when k(Q(x))=1, the capacity term disappears when taking the derivative of the present value function (8). Therefore, in the given problem set-up, an investor becomes indifferent to capacity choice if target production performance can be achieved. The same conclusion holds regardless of the type of the investment cost function.

## Footnotes

1 Note that we make the assumption (by Eq. (3)) that the adjustment of the subsidy happens in continuous time. However, in reality the adjustment happens on an annual basis. This leads to a tracking error, the effect of which on the investment decision can be considered negligible.

## References

[1] Dixit A.K., Pindyck R.S. (1994). Investment Under Uncertainty.

[2] Kozlova M., Fleten S.-.E., Hagspiel V. (2019). An alternative design of renewable energy support: investment timing and capacity choice under the Russian capacity mechanism. Energy.

[3] Boomsma T.K., Meade N., Fleten S.E. (2012). Renewable energy investments under different support schemes: a real options approach. Eur. J. Oper. Res..

[4] Vogstad K., Kristoffersen T.K., Pardalos P., Rebennack S., Pereira M., Iliadis N. (2010). Investment decisions under uncertainty using stochastic dynamic programming: a case study of wind power. Handbook of Power Systems I.

